# "The Hospital" Nursing Mirror

**Published:** 1900-02-24

**Authors:** 


### "The Hospital" Nursing Mirror.

Being the Nursing Section of "The Hospital."

[Contributions for this Section of "The Hospital" should be addressed to the Editor, The Hospital, 28 & 29, Southampton Street, Strand, London, W.C., and should have the word "Nursing" plainly written in left-hand top corner of the envelope.]

### Notes on News from the Nursing World.

### MORE NURSES FOR SOUTH AFRICA.

On Saturday the following nurses, we are officially informed, will embark for South Africa: Nursing Sister L. W. Tulloh, of the Army Nursing Service; and Nursing Sisters R. J. Briggs, E. M. Chamberlain, E. M. M. Howard, J. Lovett, M. B. Portwee, E. M. Rowley, G. G. Syles, and H. Whiteford, of the Army Nursing Reserve. The nurses attached to No. 8 General Hospital sailed in the "Dunvegan Castle" on Saturday. In addition to Miss E. Holland, superintendent, there are 19 sisters of the Army Nursing Reserve, namely: Misses M. S. Barwell, E. M. Bickerdike, A. A. Bowles, A. Brooke, V. H. Buchanan, R. M. Bullock, R. M. Carr, E. E. Coutts, C. M. Friend, M. L. Harris, L. D. Hills, A. Hilson, F. Holmes, M. B. King, A. Knaggs, J. E. Mount, J. A, Ormerod, L. A. H. Seligmann, and A. L. Walker. Miss Trew, matron of the Royal Cornwall Infirmary, who is now attached to the Army Nursing Reserve, has also gone to South Africa. Miss Frances Leng, who is sister in charge of the men's wards at the infirmary, will take the matron's place in her absence.

### THE TRAINING OF THE NURSES.

Miss Marian Sabine Barwell was trained at Leeds Infirmary, and was subsequently sister at Cardiff Infirmary. She joined the Army Nursing Reserve three months ago, but had only been attached to the Military Hospital, Devonport, for a fortnight, when she was ordered, at three days' notice, to go to South Africa. Miss Alice Anna Bowles was trained at the London Homœopathic Hospital for three years, and was afterwards night superintendent and attached to the private nursing staff. From August, 1892, to February, 1893, she was pupil and midwife at the City of London Lying-in Hospital, and then night superintendent. From July, 1894, to November, 1898, she was one of the Queen's district nurses, and from that time she has been one of the private nurses in connection with Princess Christian's Trained Nurses' Home. Miss Violet H. Buchanan was trained at the Central London Sick Asylum, Cleveland Street. Miss Catherine Mary Friend was trained at the South Devon and East Cornwall Hospital, Plymouth, where she was successively staff nurse, private nurse, and sister. From 1896 she has been engaged in private nursing. Miss Amy Knaggs was trained at Radcliffe Infirmary, Oxford, and has since been assistant nurse, staff nurse, and sister at Leicester Infirmary. In 1897 she joined the Army Nursing Service. Miss Louisa Watson Tulloh was trained at Crumpsall Infirmary, Manchester. She has since been private nurse at the Sarah Acland Home, Oxford, and the Holland Institute, Nice, entering the Army Nursing Service in September, 1887. She was in Egypt from December, 1888, to March, 1894, and was stationed at Cairo and Alexandria, going on active service through the Nile Campaign of 1889. In 1897 she received the decoration of the Royal Red Cross. Miss Henrietta Whiteford was trained at the London Hospital, and has since been sister at the Civil Hospital, Gibraltar, and St. Saviour's Infirmary, Dulwich; and temporary matron at the Memorial Hospital, Paulton, and the Seamen's Hospital, Greenwich. From November, 1889, she has been engaged in private nursing. In 1897 she was awarded the Greek medal, and in the same year that of the Maidstone Typhoid Epidemic. We regret that in consequence of the failure of the other nurses to send particulars of their training to "Burdett's Official Nursing Directory," we cannot give any information about their experience.

### THE NURSES OF THE "PRINCESS CHRISTIAN" HOSPITAL.

The "Princess Christian" Hospital, presented by Mr. Alfred Mosely, of West Lodge, Hadley Wood, will start from Southampton early next week, with the following nurses on board: Sister E. C. Laurence, in charge, and Sisters M. Leng, E. Atkins, E. M. Fisher, D. A. Snell, and F. Baker. Miss E. C. Laurence was trained at the Hospital for Sick Children, Great Ormond Street, and Guy's Hospital, where she has been night sister since March, 1896. Miss Florence Baker was trained at Guy's Hospital. Miss Minnie Leng was trained at Guy's Hospital. Miss D. A. Snell was trained at the London Homœopathic Hospital, where she was subsequently sister.

### DEPARTURE OF THE NURSES OF THE YEOMANRY HOSPITAL.

The nurses of the Imperial Yeomanry Hospital had a splendid send-off on Saturday. The platform at Waterloo was crowded in the morning with doctors, nurses and medical students anxious to wish them "God speed." The train for the "Guelph" was split up in two sections, the first taking a number of soldiers and a machine gun; the second nurses and ward-maids. Lord and Lady Georgina Curzon, with other members of the committee, joined in the adieux. A telegram has been received from the honorary civilian director in South Africa to the effect that the Commander-in-Chief has approved of the site of the Yeomanry Hospital being at Deelfontein Station, 29 miles south of De Aar. This is considered by the authorities to be by far the best location in the district, and it is well sheltered by a range of hills from the prevailing winds.

### COLONIAL NURSES AND THE WAR.

It will be recollected that some weeks ago we endorsed the request of the nurses of Australia that an effort should be made to utilise the services of a few of their numbers in South Africa. Since then a daily contemporary has put forward the statement that nearly all the applications of colonial trained nurses for appointments have met with official refusal. This is a misconception of the facts of the case. It has not, perhaps, been found possible, neither would it have been judicious, to have accepted every application from nurses already at the Cape. But in many instances their services were gladly utilised. Thus, out of four nurses who were obliged to leave Johannesburg when the war broke out three were, within a couple of months, asked to assist at the front, and another nurse, who was then at the Cape, writes:—

"On December 23rd I was asked at a few hours notice to start for home with a transport of sick and wounded. According to instructions, I was on board by ten a.m. in order to assist in receiving the wounded and putting them into their respective berths. About a dozen poor fellows were sent aboard on stretchers. It is pleasant to record that with one exception all were able to walk about before we reached the end of our voyage, which took twenty-six days. There were four nurses, and under the doctors' supervision we had each a number of the worst cases put under our charge. Never more than two were put in a cabin, a piece of thoughtfulness for the men's comfort much appreciated. Most of our cases were bullet wounds and shattered bones. Many of them had had narrow escapes. A Guardsman had a Mauser bullet which penetrated his side and came out at the elbow without injury to bone or joint, but cutting the nerves so that his fingers were powerless. A man who had a narrow escape got a bullet on his back at the right side of the waist; it passed right through him, under the spine, coming out at the left side. All that was to be seen was a puncture the size of a pencil end where it entered, size of a pencil point at exit. Another man had a bit of skull smashed out, which he sent home to his best girl to be polished and made into a brooch!"

It is only fair to the authorities that proof of this character should be afforded that nurses on the spot have not been prohibited from taking a share in nursing the men wounded in their own colony.

### AMATEURS AND SIR WILLIAM MACCORMAC.

WITH respect to the amateurs in South Africa, a high authority who has just returned from the seat of the war, discussing the injurious effects of untrained nursing with a correspondent, said:—"At the base hospitals, ladies, whose nursing qualifications are best described as nil, are clamorous to don apron and cap and help the doctor and nurse. Day by day these misguided damsels have to receive the required check, and, moreover, to have their repeated bedside visits put an end to. These had become deleterious on the score of oft-disturbed slumbers, and also through the thoughtless administration of tempting, but harmful delicacies. No more sorry credentials of their nursing efficiency could these ladies offer, perhaps, than their perpetual disregard of the diet chart that is to lure poor Tommy back to health and strength. In the interest of their patients, and despite all outcry, visitors have been debarred admission to the wards, and their gifts are by order placed upon a table which for the purpose has been put at the hospital entrance. It is only right to add that this drastic measure is in the main attributable to Sir William MacCormac himself."

### NURSING IN MILITARY HOSPITALS.

IN a letter to the *British Medical Journal* Surgeon-General Hamilton protests against the statements of Dr. Groves in his report to the British Medical Association, which was quoted in our columns three weeks ago. He says, "It is difficult to understand how such statements could have been written by anyone having the smallest knowledge of the subject," and expresses his surprise that the Parliamentary Bills Committee should have accepted "a report as full of inaccuracies as it well could be." Nevertheless, we have our doubts, and do not feel by any means convinced that the nursing in military hospitals is quite as satisfactory as some people believe. For example, the special correspondent of *Guy's Hospital Gazette*, writing under date of December 20th, 1899, from No. 1 General Hospital, Wynberg, says:—

"The strain especially falls on the wretched orderlies of the R A.M.C., who are on night duty every other night, besides being on day duty from five a.m. to five p.m. every day. Night duty with them means two hours on and two hours off through the night, the duty-hours being spent, as a rule, in visiting each of three huts in turn, while the off-hours are spent in trying to sleep on tables and benches, or on the ground round the guard-tent. Naturally this kind of life leads to festivity, as an antidote, whenever the orderlies get a free evening, and with confinement to barracks or cells for a few days, a good deal of discontent as a result. The whole system is bad, and is due not to the exigencies of war, but to the exigencies of a slowly-developed, hide-bound, almost mathematical system, clogged with petty trivialities and hampered at every turn by an excess of officials, official returns, and all the paraphernalia of red-tape. The R.A.M.C. people feel this as much as ourselves; indeed, it is the root of their discontent. It must all be changed, but now is not the time to change it. We must make the best of existing conditions during the war, and hope afterwards for a thorough, an impartial, and yet a kindly critical investigation into the whole subject."

### THE ARCHBISHOP OF CAPETOWN'S WARNING TO VOLUNTEER NURSES.

THE Archbishop of Capetown, who might be supposed to regard the Kilburn Sisters with some favour, writes from Bishopscourt, Claremont, Cape Colony: "It is in my judgment a great mistake for the Kilburn Sisters to come out to nurse the sick and wounded soldiers without the consent and approval of the War Office authorities. They may very probably find that they have come in vain." If the Kilburn Sisters find that their journey is futile, they will not derive much consolation from the Archbishop's presumption that, "unless they are excellent and highly-qualified nurses they would not have undertaken such work." We commend the Archbishop's warning to the silly people who are writing to the *Morning Post* on the value of volunteer nurses with "a little training" who are willing to proceed to South Africa at their own cost. There are plenty of volunteers of this class on the spot, but, to our own knowledge, want of training has been found to mean not only lack of necessary experience, but lack of power to bear the strain of hard work. A few of these volunteers were allowed to do a little amateur nursing when the staff was temporarily inadequate, with the result that in a fortnight they returned to their homes on the invalids' list themselves.

### WOMEN NURSES IN THE AMERICAN ARMY SERVICE.

STRIKING testimony is afforded by American soldiers as to the value of the Army Nurse Corps in the United States. Dr. Anita McGee quotes the following opinions of "enlisted men":—

"A sergeant of the 17th Infantry, who has seen twenty-nine years' service and who recently returned from Manila, where he was sick in the hospital, says: 'God bless the female nurses and those who sent them. The only trouble is that there are not nearly enough, and they are needed at night as well as day, since it is during the night that the greatest number of patients die.' Another sergeant from the same regiment, now in the States, says: 'Could the boys at the front have even the army rations prepared as they are in the diet kitchen and given to them when first attacked with bowel trouble, it would soon pass away. The

Hospital Corps men do all they can; but they do not know how, and many die at the distant posts in the Philippines for the want of a little care and properly prepared food.' Another non-commissioned officer, stationed at Columbus, Ohio, says: 'The female nurse has worked a grand transformation here, and not only here, but wherever she has gone; and if the men and officers of the line (with but few exceptions) had to decide the matter there would be no question of their remaining.'"

Dr. McGee says that these and similar remarks were noted by one of the nurses now in the service, and that like testimony could be gathered at other hospitals. She adds that "the officers and men of the line are the undoubted friends and supporters of the army nurses." Evidently, too, the military authorities in America share the views of the officers and men. In fact, they seem much more disposed to recognise the value of women nurses, and to turn them to account, than the military authorities in this country.

### THE TITLE OF SISTER.

The Bristol Board of Guardians have taken a step which will certainly not tend to render it more easy for them to obtain the services of good nurses. They have decided "to get rid of the term sister among their nurses." A Bristol paper, commenting with approval upon the decision, says "the term belongs properly to those conventual bodies which are bound together by religious vows of mediæval origin, and therefore suggests that the nurses are something different from what they really are." This will, indeed, be news to the nursing world, and to the authorities of the great hospitals in the country. We utterly deny that conventual bodies have any prescriptive right to the use of the titlt of sister. It is as appropriate for the hierarchy of the nursing profession as it is for ladies who belong to a purely religious society. Both are members of a sisterhood whose aim is the amelioration of the human race; though the attempt to substitute for the designation of "sister" that of "head nurse," which the Bristol Guardians have initiated, will be resisted, not only because of its appropriateness, but also because it is convenient and comprehensive, at once marking the status of a member of the profession who has attained a dignity beyond that of a staff nurse. The authorities of the Royal Children's Hospital in the same city clearly do not intend to follow a bad example, for, as will be observed in another column, they have juse appointed a new sister.

### THE HOURS OF ASYLUM ATTENDANTS.

At the meeting of the London County Council on Tuesday a discussion took place on the report of the Asylums Committee respecting a petition presented so far back as last June from about 700 attendants at the asylums under the jurisdiction of the Council for a reduction of their hours of duty. The committee reported that they could not see their way to reduce the hours, but we are glad that the Council requested them to consider the matter further. The result of second thoughts will, it may be hoped, be that a change will be speedily made. Several members of the Council expressed the opinion that, "even if a decrease of the hours of labour would involve extra expenditure, the money would be well spent." We entirely agree with them. As Mr. Lawson said, it is monstrous that the attendants should work 14 hours a day. Only last week we referred to the fact that the hours of mental nurses generally are considerably too long; and if the London County Council will set the example of diminishing them to 10, provincial authorities will doubtless in due time follow it.

### THE BLAIRGOWRIE NURSING CASE.

So far, Miss Christina Jackson has scored in her contest with the Blairgowrie and Rathay District Nursing Association. Lord Stormonth Darling has decided that the case must go to a jury. It will be remembered that Miss Jackson claims considerable damages for charges against her which she avers were "false, calumnious, and injurious to her, and were made recklessly, maliciously, and without probable cause, and with the meaning intended to be conveyed that she was unfit to be a nurse." She also alleges that she has since been unable to obtain employment as a nurse, and is now engaged as a stewardess on an Atlantic liner. The question of a report said to have been sent to and inserted in a nursing paper is among the issues involved.

### SHORT ITEMS.

The London committee for establishing nursing homes at Madeira for the sick and wounded from South Africa have sent an officer to report upon offers received from that island, with the result that 72 beds are now available at that station. After consultation with Dr. Grabham it has been decided to receive the majority of the invalids who may be sent thither at one of Mr. Reid's houses in the environs of Funchal, and to utilise Senor Machado's beautiful villa for the smaller number of patients requiring treatment at a much greater elevation.—Lady Castlerosse has co-operated with several of the ladies of Killarney with the object of procuring a Jubilee nurse for the sick poor of the district.—Sister H. A. Mills, of the Indian Army Nursing Service, stationed at Rawal Pindi, has been granted six months' special leave to England. This is a rare concession to the ladies of the I.A.N.S.—Miss Alice Gilmore, serving in West Africa under the Colonial Office, has been gazetted to the Indian Army Nursing Service, and leaves for a tour of service in the Bengal command immediately.—The St. George's-in-the-East Board of Guardians find it so difficult to obtain nurses that they have asked the Local Government Board to consent to them advertising for probationers.—Miss Higgs, one of the nurses of the Imperial Yeomanry Hospital, wishes to state that she was trained at North-West London Hospital for three years, and remained there until November, 1899, when she was appointed to the Royal Hospital, Waterloo Road, as out-patient staff nurse.—The medical superintendent at the Camberwell Infirmary having reported that there are still seven vacancies on the nursing staff, the guardians, at his suggestion, have agreed to appoint six further probationers.—The Queen Victoria Nurses and others from various hospitals in Wolverhampton were invited by Mr. Van Biene, the lessee of the Grand Theatre, to hear him in "The Broken Melody."—A grand concert was given on February 16th at the St. George's Vestry Hall, E., by Nurse Marie Harrod, M.R.B.N.A., and Queen's Nurse, and Miss Timbrell. The hall was well filled with a large and appreciative audience. The proceeds of the concert amounted to £20. Two pounds eleven shillings and sixpence was forwarded to the Absent-Minded Beggar Fund, and the rest, £17 18s. 6d., will be sent to H.R.H. Princess Christian.

### Clinical Charts and How to Keep Them.

By a Charge Nurse.

POINTS FOR PROBATIONERS.

No one will be long in hospital before becoming acquainted with the temperature chart. They are ruled sheets of paper, and their primary object, for recording the temperature, pulse, and respiration. But they should be still more. They are meant to be a simple and concise summary of the treatment and developments throughout the course of a disease, whether medical or surgical. Much depends on the way they are kept. The idea of writing on the subject suggested itself to me through having at different times staff nurses, good nurses at practical work, but still not clever in recording the various points really essential on the one hand, whilst excluding all extraneous matter on the other. Then, I thought, it might to some extent overcome the difficulty and be serviceable to new beginners, if I were to describe their uses as simply and concisely as possible.

There are various sized charts, some varying slightly in the arrangement of their spaces for the several entries. I should not like to say which is "the best," for no doubt every surgeon, like every mother with her baby, thinks his own design the best. I will only say that for my own use I prefer those which have the spaces for the day of the month and the day of the disease at the top of the chart, and those for the stools and urine at the bottom. I also like charts which have spaces for reaction, sugar, albumen, or any other abnormalities in the urine, in addition to the one for recording the number of ounces passed in twenty-four hours. Of course these items are of minor importance, and each nurse must use the chart provided by the authorities for whom she may happen to be working.

Now regarding the keeping, the patient's name, age, disease, &c., will, no doubt, be filled in by the sister; but I am going to suppose your doing that duty yourselves. Write as neatly and legibly as possible. The first point I would impress on all new beginners is accuracy. I do not mean to infer that anyone would report "Two pills four-hourly" if only one had been given. I refer to date. If an enema is ordered one day and you make your chart up next morning, do not enter the enema under the date of that day instead of the day on which it was given. Then, again, if two aperients are prescribed, one to be given at night the other in the early morning, do not put them both down for the same day. Calomel grs., eight p.m.; mist alba ℥ii., four a.m., should be written. These are points which must be observed if the chart is to be a reliable guide to the doctor. When you are keeping a four-hourly chart, suppose your times are four, eight, twelve. What happens after four and before eight is put down under eight; that after eight and before twelve under twelve, and so on through the day and night.

My next point will be some of the details which it is always as well to note on a chart. Of course, you know all cases have not the same symptoms, and I would not have you think that the few points I am noting are the only ones to be observed in every case. I am simply touching on a few diseases as a guide and to encourage you to cultivate the habit for yourselves. As you become better acquainted with disease and the symptoms to be watched for in every case, you will understand what is best to chart and what is not. Always keep in mind the necessity of not having your chart too crowded. The chart is not a case-book, nor is it to save the nurse from taking notes from day to day.

When you have made out a chart for a new patient, and have charted the admission temperature, it is always as well to note, "on admission, three p.m.," as the case may be. If it is pneumonia, and the sputum rusty, note the same. For such a case you will, of course, be keeping a four-hourly chart. Then there will be more room for notes. When delirium comes on it should be noted under the proper time and date, and when it has passed off, "delirium subsided" added, and if any sleep, how much. And here let me ask you, when on night duty to observe closely at what time and for how long together your patients sleep, and be able to give a clear account to your sister in the morning. Do not fall into the habit of saying "Slept fairly well," "Slept a little." Such expressions are too vague. If sponging takes place to reduce the temperature, simply say tepid or cold sponged, whichever it may be. The temperature should be taken after the sponging and charted, with the note "after sponging" added. Should the patient be packed, the time he is put in and the time taken out must be recorded. "In cold pack from three p.m. to six p.m." is sufficient. The first appearance of herpes about the mouth should be noted.

For phthisical patients you may have a special chart, with spaces provided for the amount of sputum, weight, chest measurement, hæmoptysis, &c. Measuring the sputum, hæmoptysis, vomit will always fall to the nurse, and must be entered correctly in their proper spaces. Weight daily or weekly, and chest measurement may also be left for the nurse to take and chart. Observe and note the time when perspiration is greatest, and also if it increases or decreases, whether there is dyspnœa or cyanosis. The first sign of œdema of the feet so common in advanced cases must not be omitted. Many other symptoms may occur and must be noted as they appear.

The tongue in most fever cases will be "thickly coated." Epistaxis is common in typhoid fever, and often hæmorrhage from the bowel. When it is mixed with fæces or a measure is not to be had make some allusion to the amount by remarking "slight" or "copious" as the case may be. The character of the stools must be put down, and also when the first "formed" stool is passed. In charting the stools of typhoid where diarrhœa is present keep to what I have before said relating to time. Count from twelve midnight to twelve midnight as one day. I always have a little book for my night nurses to put down the time the bowels act of a typhoid patient, and then, when making up the chart next morning put in those before twelve on one date and those after on the next. It is very essential to know how often the bowels act in each twenty-four hours. If any drugs are being given, such as salol, say, salol grs. vi., 4 hrs., or pil plumbii opio i. 4 hrs.

Any tenderness, abdominal pain, and distension are important notes to make, and also the rash or spots as they appear. Write two spots, more spots, many spots, if such is the case, from day to day. If the patient has constipation each enema will have to be put down. Some notes may be abbreviated—enema glyc. will answer the purpose. Make a note when the first stool is passed in the natural manner. When a soap and water enema is given, write enema (simple). In diabetes the nurse has to measure the amount of urine daily, report the quantity, specific gravity, and the amount of sugar. The first sign of a moist skin and an abatement of thirst it is as well to remark upon. In any case, if the pulse is thready, intermittent, weak, or irregular, make a note of it, if possible, at the bottom of the chart by the pulse.

All hypodermic injections should be put down: Hypo. Inj. Pilo-Carpin gr. ¼, Hypo. Inj. Morphia gr. ¼. In pleuritis with effusion "Paracentesis Thoracis," or aspiration, may be performed, perhaps several times. Write "Aspirated" or "Paracentesis Thoracis" Fluid oz., after carefully measuring the quantity drawn off.

From what I have said about medical cases I think you will have some idea of what is required of you, so I will now pass on to surgical ones. Suppose you have just made out a chart for a patient on whom an operation was performed at ten a.m., write "Operation ten a.m." After the patient has recovered from the effects of the anæsthetic the temperature should be taken and charted, with "After operation" written by the side. When hæmorrhage occurs it should be noted. Write "Dressed, first time," when such has taken place. If any sutures have been removed, say how many. When a drainage tube has been used, note when it is discarded and say when the wound is quite healed.

Much sickness after an operation should be recorded. If an incision is made, perhaps in an abscess, or several incisions, either all at one time or at various intervals, enter each under their proper date. Make some reference to the amount of discharge and the frequency of dressing a wound by remarks such as "Dressed daily," "Dressed twice daily," "Fomented (Boric) four hourly," "Discharging freely," "Thick pus," "Very little discharge," "Discharge offensive," or "Not any discharge."

My last point is "neatness." This is least important, but still, I hope, nevertheless, that all will try to cultivate it. It is very annoying to see a chart blotted, or thick lines between some dots and thin ones between others. It is not simply a question of time, but a question of habit. If you are determined I am sure you will be able to make up your chart both quickly and neatly, and without encroaching upon any of the time which should be devoted to the patient's nourishment or any other attention.

Try to make your dots indicating the temperature uniform, whatever size they may be. Some nurses like a very large dot. I think they should vary according to the size of the chart. Do not, if using a very small chart, with the points between the degrees not marked off, make a very large dot so that the doctor could not tell without asking whether the temperature is 100·2 or 100·8. Let the lines connecting the dots be fine and straight. If you are keeping the chart of a phthisical patient, at that stage of the disease when there is the evening rise and the morning fall, it will entail the drawing of very long lines. In that case it is better to use a ruler.

For making entries and lines a mapping pen is useful. The chart must never be too crowded for the temperatures to be read easily. Every item should not be recorded, only principal ones, and if you will only bear in mind what I have said, and observe also how charts are kept in your training school, I am sure you will not be liable to make many unnecessary entries or mistakes. We cannot see the practical work of all hospitals, but many of us may have opportunities of learning from others whom we come in contact with. I am indebted to some of the doctors under whom I have worked for many valuable hints. Some doctors you may meet who like very few entries; in that case, all I can say to you is to treat with deference and give implicit obedience to those who are officially your superiors.

### To Nurses.

We invite contributions from any of our readers, and shall be glad to pay for "Notes on News from the Nursing World," or for articles describing nursing experiences, or dealing with any nursing question from an original poi t of view. The minimum payment for contributions is 5s., but we welcome interesting contributions of a column, or a page, in length. It may be added that notices of entertainments, presentations, and deaths are not paid for, but, of course, we are always glad to receive them. All rejected manuscripts are returned in due course, and all payments for manuscripts used are made as early as possible at the beginning of each quarter.

### Nursing in the Orkneys.

By a Sister.

Only a very limited number of the dwellers in the south are aware that the distance between Orkney and the mainland is but seven miles, though of the beauty of the islands already so much has been said and sung that I need not touch on that point. My object is to deal with the great need of a thoroughly organised nursing staff throughout the county.

The population of Orkney is 30,453. We have about 75 churches, with the same number of ministers; therefore, we ought to be religious. We have about 60 schools, with at least 160 teachers; therefore, we ought to be educated. We have only 17 doctors, and not one qualified nurse; therefore, we ought to be unhealthy. Or, is this dearth of medical men and women due to the dearth of disease? The invigorating influence of the seabreezes, no doubt, does much to "harden" Orcadia's sons and daughters, but here, as elsewhere, disease and death are not unknown. Why, then, have we so few medical men? Because a doctor, his wife and family, require £500 a-year. And there are so few invalids here that in many parishes a doctor could not raise £50, that is to say, if only those who require his services contribute to his salary. In some parishes a certain amount is guaranteed. This is raised by general subscriptions. In addition, the doctor is paid for his attendance and medicines by those who employ him.

Though the doctor may be many miles away, as long as the distance can be covered by horses, there is no serious drawback. But if the ocean intervenes the case is slightly different. North Ronaldshay, the most northerly of the group, with a population of 500, is dependent on the doctor from the neighbouring island of Sanday. The distance between those places is only three miles, yet at times the Sound is so stormy that it is impossible for any craft to cross it may be for days at a stretch. How terrible must it be for the inhabitants of this island, when sickness comes, to be so entirely cut off from skilled advice. And North Ronaldshay is not the only island where this state of matters exist. Now comes the question, "What can be done to remedy this?" Supply these outlying districts with trained nurses! Nurses may not be perfect, and are not always able to cope with the ailments they have to encounter; but there are nurses, wise, capable, and cool, who know more about the real practical side of treatment than some doctors in practice to-day.

There is a prevalent idea in some parts that because in hospital life a nurse is under the doctor's jurisdiction, if he is not at hand to lean on she is utterly useless. True, there is always a doctor within call, but how much has every young resident to thank his nurses for in the days when he was "green"? It would be treachery to depreciate the doctor's worth, and presumption on the part of any nurse to willingly take upon herself a doctor's responsibilities, but most nurses have enough common sense to obviate this. Nor do I think there is danger of the Orkney medical practitioner objecting to the innovation of nurses. The relation between doctors and nurses all the world over may fitly be described as "A Mutual Admiration Society."

Would nurses from the south be willing to spend their lives in Orkney? Even in North Ronaldshay we have a minister and a schoolmaster, both of whom were brought up in more populous regions. Why, then, should nurses not be content with a life of peace far from the madding crowd? Kirkwall, our capital, is blessed with five doctors, still there is urgent need of a district nurse; and life in Kirkwall is ideal. It is honoured with the name of city, yet enjoys all the advantages of country life and all the talent and culture of a West-end drawing-room. It may be comforting to those who shrink from taking up work in our midst that it is possible to live in Orkney and still keep abreast of the times. Even here THE HOSPITAL and *British Medical Journal* are not unknown.

But nurses cannot live on fresh air and literature alone. Where would the salary come from? The Home Mission of the Church of Scotland in cases considered suitable by the committee are willing to pay a sum of £50 yearly for a parish sister. In Orkney a nurse would be better suited than any other for this post. The parish might surely raise another £20, and then everybody needful, irrespective of creed, would be supplied. Might not the County Council, whose duty it is to look after the general welfare of the people, take up the matter?

The Orcadians are a peculiar people. Cradled in their ocean-swept homes, is it strange that to those who know them not they appear hard, unimpressible, and immovable as the rocks which guard their coasts? To those who are privileged to read them aright they are sincere in friendship, unflinching in duty, exact in business, and honourable in their dealings with their fellow men. They have been accustomed to do without luxuries, and they miss them not. But is a nurse a luxury? Methinks she is a greater necessity than a Sunday bonnet, in times of sickness at least. And if Orcadians only had an opportunity of testing a nurse's usefulness, there would be no community more ready to prove their appreciation of her services.

### The Nurses' Bookshelf.

[We invite Correspondence, Criticism, Enquiries, and Notes on Books likely to interest Women and Nurses. Address, Editor, THE HOSPITAL, (Nurses' Book World), 28 & 29, Southampton Street, Strand, London, W.C.]

MENTAL NURSING. By WILLIAM HARDING, M.D., M.R.C.P., Medical Superintendent Berry Wood Asylum, Northampton. (London: Scientific Press, Southampton Street. Pp. 91. Price 1s.)

THE preface to this little book informs us that the greater part of its contents has already been published by the Scientific Press under a similar title, and that two editions being exhausted it was thought desirable to revise, condense, and issue the new book at a lower price and in handier form than the old one. In its new guise the work has lost none of its attractiveness. Indeed, from some points of view, it is distinctly improved. A preliminary chapter deals in a satisfactory way with the general duties of a mental nurse, and various hints are given as to the qualities, mental and physical, which every woman should possess who undertakes the difficult and not always pleasant duties of nursing the insane. The various forms of insanity are described, but Dr. Harding has wisely refrained from burdening his pages and confusing the nurses' minds with long drawn-out classifications of mental disease; classifications which mean little to the physician, and nothing at all to the nurses. The chapter on epilepsy, although short, strikes us as being very happily expressed. The trouble which these patients give the nurse, whether in asylum or in private practice, is clearly described, and the method of dealing with them clearly pointed out. Dr. Harding says, "they (the epileptics) often display deep religious feeling, and at the same time are untruthful, given to stealing, prone to making false charges, and are always ready to quarrel. With them the blow generally comes before the word. A fancied insult is frequently the only reason assigned for a savage onslaught upon an unoffending and harmless bystander." Other sections deal with the nursing of mental cases in their own homes (and as Dr. Harding truly says, this course should be followed in many varieties of insanity); with the administration of medicines; with forcible feeding; how delusions should be dealt with; the management of suicidal cases, &c. For this latest addition to the "Burdett Series of Text Books on Nursing," we venture to predict an even greater measure of popularity than the author's former volumes on mental nursing. It will prove equally valuable to the asylum nurse and to the mental nurse engaged in private work.

NURSING IN DISEASES OF THE THROAT, NOSE, AND EAR. By MACLEOD YEARSLEY, F.R.C.S. (London: The Scientific Press. 1899. Price 2s. 6d.)

THERE are many circumstances in which this short manual will be found very useful. A training school must be very well managed to enable every nurse to obtain a practical familiarity with the somewhat special work which is done in the aural and throat departments, and thus there are probably many nurses, and we are not sure that there may not be a considerable number of medical students to whom much that is contained in this book will be novel as well as interesting. The book commences with a short account of the physiology of the parts in question, namely, the throat, the nose, and the ear, after which comes a chapter of general instructions regarding the instruments used in diagnosis and treatment, and the methods employed in what may be called minor treatment, the application of remedies, syringing the ears, the use of sprays and douches, and the giving of inhalations. After this we have a short account of the various operations which may have to be done upon the ear, giving in most cases the instruments which will be required and the sort of dressings and lotions which may be wanted. The chapter on the after treatment of these operations will be very useful, for however much a surgeon may look after things for himself at the time of his operation, the nurse may easily upset everything if she does not know exactly what to do. The same division of subjects is found under the head of diseases of the throat, and a special chapter is devoted to the subject of laryngotomy and tracheotomy. Altogether the book will be found very practical and instructive. It is full of illustrations depicting the various instruments mentioned in the text, so that no nurse who reads it carefully need be at a loss as to the meaning of the terms used.

HOME NURSING. By SISTER GRACE. (Published by C. Arthur Pearson, Limited. Price 1s.)

SISTER GRACE has succeeded in compiling a valuable little handbook, which "Isobel," of *Home Notes*, has edited. The advice as to dress and manner is excellent, and so is that relating to the care and ventilation of the sick-room, the making of "staying" fires, &c. The instructions as to nutrient enemas are less accurate; the "pear-shaped syringe" is not now looked upon as the best vehicle for administration, nor would Sister Grace's limitation of the quantity to two ounces coincide with the instructions given by some physicians at the present time. We must also take exception to the indefiniteness of the precautions advised in the treatment of an epileptic. The direction to "place a pad in his mouth," if carried out literally, might result in the patient's death at the hands of someone who was unaware that the separation of the poor man's teeth was all that was necessary to prevent injury to the tongue. Sister Grace gives her readers some capital hints on the disinfection of rooms and clothing, and the careful isolation of cases of infectious disease. The little volume is so sound in its teaching on most points that the authoress may well be excused for the trifling errors of which we have felt obliged to take notice.

### Antiseptics for Nurses.

DISINFECTION BY HEAT (*continued*).

In Bredenberg's steam disinfecting apparatus the arrangements are very similar to those in Schimmel's stove, and in it also no provision is made to do more than partially dry the clothing afterwards; and, in fact, the Continental authorities do not seem to attach as much importance to this point as we do in this country. It is made in two forms, as a small stove in which the steam-generating apparatus is as simple as possible and heated by a fire below. The water is contained in a dome-shaped receptacle at the bottom, which has to be filled by hand through a funnel instead of being self-filling, and consequently it requires much care and supervision that the water shall not all evaporate, and the receptacle is not deep. The only way of shutting off the steam is by withdrawing the fire, and this has to be done after each stove-full of clothing has been disinfected. To secure circulation of air there is a small door provided at the top of the apparatus, and when opened there is a certain amount of circulation of air owing to the heat in the clothes and in the walls. The steam generated in the receptacle is carried upwards and discharged at the top, passing downwards amongst the clothes, and as it condenses is collected at the bottom and returned by a pipe into the receptacle. In the larger and more elaborate apparatus steam is generated in a separate boiler, and the air is heated previously to letting the steam escape into the chamber. The only means of securing circulation of air afterwards is by opening a door at the bottom of the chamber, so that the air can enter, pass upwards, and escape again at the top by means of the exhaust pipe. Before use the apparatus must be thoroughly warmed by hot air for at least half an hour, and after the articles to be disinfected have been put into the chamber they must also be thoroughly heated, a process which will require at least another half-hour, probably more. Steaming the articles will require from half an hour to an hour, according to the number put into the chamber at one time, and also to their bulkiness; and, finally, they must be left for another twenty or thirty minutes to dry somewhat, for there is no pretence made of thorough drying, and the articles have to be removed to a drying-closet to complete the process. Consequently the whole process of disinfection is a very lengthy one, occupying a large amount of the time and attention of the attendant in charge of the apparatus. However, it is claimed for all these German disinfectors that they are much cheaper than the English ones in the first instance, since they are made to work at low pressure, and the long exposure of the clothes to steam, and their consequent wetting, is not considered a serious drawback. The advantages claimed for the German steam disinfectors are: (1) The clothes are warmed by hot air at a temperature of 60 deg. C.; (2) the clothes are steamed by current steam at atmospheric pressure; (3) the clothes are partially dried, and aired again by warm air; (4) articles which are not very bulky are thoroughly disinfected; (5) the first cost of the apparatus is moderate. Such a disinfector would not be considered entirely satisfactory and efficient in this country, where high-pressure steam is recognised as more valuable and more certain in its results than steam at atmospheric pressure or than current steam. Also a more durable apparatus would be considered preferable, even if the initial cost is greater; still it would be regarded as cheaper in the end, and one, too, furnished with appliances to shorten the time necessary for the process and to save labour, which is more costly in this country than it is on the Continent. Another advantage of a high-pressure apparatus is that, when necessary, it can be used at low pressure, whereas the reverse is not the case, and an apparatus originally built for low-pressure steam cannot be used for high pressure. Another serious drawback to the use of low-pressure disinfectors is the impossibility of ascertaining the temperature within the chamber at any moment during the process without a pressure gauge, which is only applicable to the high-pressure machines, and to the fact that the efficiency of the disinfection must depend on the care and attention of the stoker. Consequently either a very conscientious attendant is necessary or unremitting supervision.

As we have already seen, disinfection of clothing is far more effectual when carried out by steam than by dry heat. Still there are some articles which cannot be disinfected by steam, and under some circumstances dry heat is required, consequently we will now consider the only hot-air disinfector that is in general use—that of Ransom. The chamber is of iron, and its floor is perforated with holes to allow the entrance of the heated air from the bottom, and outside is a casing of wood, whilst the space between the iron and wood is lined with specially prepared non-conducting and non-inflammable felt, and by these means undue loss of heat is prevented as far as possible. The necessary heat is generated by means of gas burners, which are arranged below the chamber, and are surrounded by a large iron cylinder, which prevents the dissipation of the heat. From this cylinder a tube is carried to the bottom of the chamber, and the heated air enters by means of the perforations in the floor, and as hot air has a tendency to rise, it is readily distributed throughout the disinfecting chamber. There are two ingenious contrivances in this apparatus: one is a self-acting mercurial regulator placed where the gas enters to supply burners, and which expands as the heat increases, and is so designed that when a given temperature is reached it has expanded so much that it shuts off the gas; the other is another method by which the gas can be extinguished in case the articles in the chamber should catch fire. A limb of fusible metal is attached to a valve in connection with the outlet flue at the top of the chamber, and when the temperature exceeds a given height the metal melts and the valve closes. In this apparatus the chamber is heated by currents of hot air, and Dr. Parsons found that in consequence the distribution of heat throughout the chamber was far more uniform than in the other dry air apparatus where the interior is heated by radiant heat, that is when heat is directly applied from burning coal or gas to the floor or sides of the chamber. In this latter class also the walls of the chamber become very hot, and if articles of clothing come into contact with the hot metal they are very liable to be scorched, even if they are not actually set on fire. These drawbacks do not exist in the Ransom dry air apparatus, when the chamber is heated by air which is warmed before it enters, and consequently the walls are not hotter than the air inside. Nevertheless, the difficulty remains that in all hot air disinfectors a uniform heat cannot be obtained throughout the whole chamber, but the heat is much greater in some parts of the interior than at others. In addition to the unequal distribution of heat in the disinfecting chamber must be added the difficulty of keeping the temperature at a uniform degree, and the uncertainty of the index of the temperature of the interior; also the length of time necessary to heat the interior to the required degree, the want of power to penetrate thick materials, and the greater length of time required for the disinfection to be really efficient. All these drawbacks will cause steam disinfection to be preferred to dry air whenever possible.

### Echoes from the Outside World.

AN OPEN LETTER TO A HOSPITAL NURSE.

If the progress at the seat of war continues to be as satisfactory as it is at present the Queen's gracious appeal to her old soldiers, which, it is calculated, means an increase of 45,000 men to the home army, will prove to have been hardly necessary. For it begins to look as if those who are now getting ready for South Africa will scarcely be required, and that those already there may be back again before very many months are over. But, wisely enough, the War Office authorities are not allowing themselves to be influenced by present successes. The steady flow of horses and men to Cape Town goes on just the same, and now, lest our own islands should run any risk of being left defenceless, the Queen has personally asked her old officers and men to re-enlist for a year. Money inducements are offered, and provision will be made for wives and children, and for return to civil life at the end of the year. As to the war itself, such good news as the relief of Kimberley, the flight of Cronje from Magersfontein, and the capture of an important position dominating Colenso—Hlangwane Hill—by General Buller is so rapidly spread that it is known to nearly every soul in the land. Even the children participate in the general rejoicing. It is difficult to determine who comes in for the greatest praise—Lord Roberts, for his well-laid schemes so wisely kept secret; Lord Kitchener, for his admirable work done behind the scenes; or General French, whose entry into Kimberley must have been a veritable triumphal march. Any day we may hope to hear that we have got rid of our Mafeking and Ladysmith entanglements, or that Cronje has been forced to give his pursuers battle. Meanwhile we are content to wait, grateful for the blessed change which one short week has brought about.

---

During the past week the Queen has made two gifts which have attracted attention. She has contributed £1,000 to the Indian Famine Fund—which, I am delighted to say, is mounting up rapidly, notwithstanding all that has been subscribed for the war—and a new bugle to Bugler Dunn. One cannot help sympathising with the dislike the brave little fellow evinced to being "made a fuss with"; and, instead of being proud of the fact, if I were the lady who kissed him when he got to Osborne, I should feel horribly ashamed of myself that I had treated like a child a boy who had proved that, under fire, he could behave like a man. The Queen's quiet way of behaving to her visitor must have been quite a relief to the fifteen-year-old hero, who must have begun to wonder how many embraces he was to suffer. He was, evidently, from the unvarnished account of his doings, a plucky little chap, and he will cease to regret his old bugle lying at the bottom of the river now that he has the new copper one with silver mounts which the Queen presented to him. But I expect that the person who is most delighted with the whole incident is the boy's mother.

---

Although Mr. Balfour has speedily been as good as his word and appointed a Committee of Inquiry into the Royal Patriotic Fund, I find that everyone is crying out about the weakness of its constitution. The omissions are certainly odd. They include Mr. Kearley, M.P., who is the real instigator of the investigation, though two of the Commissioners of the Patriotic Fund have been nominated; an actuary of experience, whose presence on it everybody says is a necessity; and, indeed, the number of business men is just as small as it should have been large. It does seem odd, does it not, that with men of splendid powers of organisation and the requisite leisure available, one of our busy judges and two of our most occupied members of Parliament should have been put on the Committee? The defence of the First Lord of the Treasury has by no means convinced the public that the tribunal is the best that could have been selected.

---

The suggestion made by a lady in one of the daily papers that a movement should be started to raise a "Woman's Volunteer Rifle Force" is extremely likely to meet with the ridicule which, it is only fair to say, the writer anticipated. The notion that a woman is the better, not the worse, for being able to shoot straight meets with the approval of most of us; and when the summer days come the monotony of tennis and garden parties might be varied by gatherings where "target shooting" by ladies for prizes might form the chief attraction. These to be successful would need private practice at home on the part of the competitors, and should the present martial spirit be strong enough amongst us, the innovation might "catch on" and become a fashionable craze, as archery once was. But to say that "if women were to organise and learn to handle modern weapons with skill by target practice and drill in volley firing" the result would be that "the women volunteer rifles would be a force to be reckoned with by an invading enemy" is the dream of the enthusiast rather than the prophecy of a woman of common sense. One thing alone is likely to prevent the realisation of the scheme. A special Act of Parliament would be required to legalise the movement, and I can scarcely imagine the men at St. Stephen's regarding a scheme for the organisation of fighting women with favour.

---

I do not know whether any of you were present at the service in St. Paul's Cathedral last week for the Queen Victoria Clergy Fund, but the opinion of Canon Gore, who preached the sermon, is worth quoting. After showing that St. Paul made a clear and intelligible claim for the officers of the Church to receive due payment for services rendered, he proceeded to touch upon the marriage question as regards the clergy. He did not, he said, undervalue the voluntary sacrifice of celibacy—he himself is a bachelor—but on the level of prudence and common sense he maintained that the clergy should be paid, and have a right to "be once married, and lead about a wife, even in the missionary field." Naturally his remarks have occasioned a great deal of discussion, more especially in the homes of the clergy themselves. Not only, of course, are there many rectors and vicars who have been married more than once, but four, at least, of our bishops, having lost their first wife, have taken another, and the retiring Bishop of Liverpool has been married three times. Those who are neither widows nor widowers pass over the first suggestion that remarriage is not right, but are very indignant at the idea that a wife is made to be "led about," as if she were a child or a little dog. Probably Canon Gore did not intend to insinuate any such thing; he was merely not very happy in the choice of a word. But he is very likely, during the next week or two, to be asked a few leading questions on the subject by the ladies of his acquaintance, I fancy.

### The Queen at the Royal Infirmary, Ryde.

By One of the Nurses.

Surely the children who happened to be patients in the "Queen Victoria Ward" of the Royal Isle of Wight County Hospital, Ryde, on Friday last were born under a lucky star, for on that afternoon they had the honour of a visit from the Queen herself, Princess Beatrice (president of the hospital) and Princess Victoria of Schleswig-Holstein accompanying Her Majesty. The Royal party arrived shortly before four o'clock. The Queen was received by the chairman (the Rev. W. E. Willey), the senior medical officer (Dr. Davy), and the matron (Miss G. Sked). The Royal party then proceeded to the new wing opened by Her Majesty in July last, and entered the children's ward, which was beautified with lovely flowers. The little ones were at once struck by the Indian attendant in his picturesque dress, who wheeled the Queen's chair, but their attention was soon transferred to the Queen herself, who visited each cot, talking to the children, holding their hands, and asking many questions about them. To each she gave a suitable toy. Musical punchinellos or soft, furry pussies to the babies; dolls to the little girls; engines or boxes of soldiers to the boys. The children were delighted with their gifts, and with the kindness of the Queen. One little boy of ten years old tells every one, as he shows his box of soldiers, "And the Queen she shook hands with me, she did." The sister and nurse on duty, who were the only nurses present in the ward during the visit, were introduced to the Queen as she passed. Her Majesty remarked, "How happy all the children seem." One tiny baby indeed—only ten weeks old—woke up to the discovery that it was four o'clock and feeding time, and made the fact known in very audible tones. Nurse, however, had the feed all ready, and "baby" promptly subsided, the Queen watching him enjoy his "bottle" with some interest. Another little boy, two years old, a great pet and generally called "Brother Charles," after staring solemnly at the Queen, held up his horse and said, "Gee-gee." On leaving the ward, the Royal party inspected "Sister's" room, and then passed along the corridor to the hall, where the nursing staff were assembled.

The Queen expressed her pleasure at all she had seen, especially with the Sun Room, round which she was wheeled. Unquestionably her visit and her gracious manner gave great pleasure to the inmates of the hospital and to the nursing staff. All the patients who were able to be up crowded to the windows to catch a glimpse of Royalty.

### Appointments.

Gateshead Children's Hospital.—Miss Marianne B. Dawson has been appointed Nurse-Matron. She was trained at the Infirmary, Sunderland.

Dr. Gray's Hospital, Elgin.—Miss M. S. Cameron has been appointed Matron. She was trained at the Royal Infirmary, Edinburgh. She has since been engaged in private nursing at Elgin, and as head nurse of the Accident Ward of Salisbury Infirmary.

Miss Atkinson's Convalescent Home for Children, Tynemouth, N. Shields.—Miss E. Shipley has been appointed Matron. She was trained at the Belfast Royal Hospital, and for the past three years has been sister of the enteric wards, Monsall Hospital, Manchester.

Monsall Fever Hospital.—Miss E. J. Kemp has been appointed Assistant House Matron. Her previous appointments have been superintendent of female epileptic wards, Manchester Workhouse, for three years, and assistant superintendent of Macclesfield Asylum for eight years.

### To the Nurses of the Imperial Yeomanry Hospital.

["A most pleasing feature has been the enthusiasm manifested by the whole of the nurses. There was not one out of the 700 applicants who was not keen to go to South Africa."—*See Report, February 10th.*]

Go! for the battle rages, and the moan
 Of wounded soldiers rises from the field.
Would we could give you strength, for you alone
 Can render service we would die to yield.
We do not grudge your glory or your praise,
 The thanks of heroes or the lasting gain—
The glad, sweet thought to lighten all your days—
 That you are chosen to ease England's pain.
Only remember you are but a part
 Of that great host which can but weep and wait,
With eager hands outstretched and aching heart,
 Craving the work you claim by kindlier fate.
Go! show the nations who now mock and hate
 How English women love to serve the State.

B. M. Cawood.

### Minor Appointments.

Royal Halifax Infirmary.—Miss Theodora Unwin has been appointed Sister of the male medical ward. She was trained at the Liverpool Nurses' Training School, in connection with the Liverpool Royal Infirmary.

Suffolk County Hospital, Bury St. Edmunds.—Miss A. M. Wycherley has been appointed Night Sister. She was trained at the Liverpool Nurses' Training School, in connection with the Liverpool Royal Infirmary.

Watford Isolation Hospital.—Miss E. Mansfield has been appointed Charge Nurse. She was trained at the Macclesfield General Hospital and Ladywell Sanatorium, and has since been engaged in private nursing in Southport.

Guildford Workhouse Infirmary.—Miss Alice Holding has been appointed Assistant Nurse. She was trained by the Meath Workhouse Attendants' Association at St. Peter's Home, Woking.

Strand General Hospital.—Miss Kate Elliott has been appointed Sister. She was trained at Huddersfield Infirmary, and has taken sister's holiday duty in the operating theatre and temporary charge of various wards in her training school.

Royal Children's Hospital, Bristol.—Miss Annie Fraser has been appointed Sister. She was trained for four years at the South Devon and East Cornwall Hospital, Plymouth, and for the last eight months has been theatre sister at the Bedford Infirmary.

St. Helen's Hospital.—Miss Emily Farrar has been appointed Ward Sister. She was trained at the Liverpool Royal Infirmary. For the past twelve months she has been attached to the Shaw Street Home for District Nurses, Liverpool. Miss Farrar also holds the L.O.S. certificate.

Chelsea Infirmary.—Miss Marion Clark, who has held the position of charge nurse for five years, has been appointed Night Superintendent. She was trained at the infirmary. Miss Nora Barnard and Miss Charlotte Lampard have been appointed Charge Nurses. They were trained at Chelsea Infirmary.

Hunslet Union Workhouse Infirmary.—Miss Annie K. Marley and Miss Florence Turner have been appointed Charge Nurses. Miss Marley was trained at Leeds Union Infirmary, and has been assistant nurse at Scarborough Union Infirmary. Miss Turner was trained at Salford Union Infirmary, and has since been charge nurse in the same institution.

### Everybody's Opinion.

[Correspondence on all subjects is invited, but we cannot in any way be responsible for the opinions expressed by our correspondents. No communication can be entertained if the name and address of the correspondent is not given, as a guarantee of good faith but not necessarily for publication, or unless one side of the paper only is written on.]

### THE AGE LIMIT.

"M. G." writes: I desire through your pages to thank Miss Gardner for her very able paper in the "Nursing Mirror" of February 10th in connection with the narrow age limit now imposed on the employments of women generally, but more especially of nurses. This question, if allowed to continue, will become a more and more difficult one, but it need not continue. Let every nurse be engaged on her own merits and capabilities as an individual, and without regard to the growing fashion of limiting age. After all, it is physical and mental strength which tell, and one woman at 45 may be as sound in these as another at 35. I am glad to say there is a scheme in progress in connection with the Royal British Nurses' Association for the employment of older nurses in private work.

"Thirty-Seven" writes: I beg to call attention to a case in point, illustrating the tendency to lower the age limit for matrons. In the current issue of The Hospital I find an advertisement for a matron, who must hold a three years' certificate from a general hospital, and have had experience in ward management and housekeeping. Age from 27 to 32. Both limits are surely unreasonable. To have passed through the three years' curriculum and the supplementary experience required at 27 is barely possible, while the exclusion of all women over 32 is a cruel hardship. Applications are to be addressed to a clergyman, well known in East London for his philanthropy. How far he may be individually responsible I know not, but the fact is to me significant. In many ways touching the great labour question the Church has not been guiltless. Too often with her right hand she has oppressed the worker, while with her left doling out the charity which saps self-reliance in the recipients. I would beg the advertiser, priest and teacher though he be, to consider that no act is isolated in its effects. The influence of this will be in the direction of reducing the age limit and so increasing the hardships from which women workers suffer.

### THE MONOTONY OF PRIVATE NURSING.

"A Hater of Cant" writes: I confess to having some sympathy with "An Admirer of Florence Nightingale." I do not see that she need be the terribly unwomanly creature that self-styled "Sympathy" considers her, merely because she grumbles a little at having to nurse women with imaginary instead of real ailments. Without describing myself as a "nurse of wide experience under our most distinguished London doctors," I may say I have had some experience in private nursing. I certainly did not find all my cases monotonous—far from it—but I have occasionally had a patient for whom the best prescription would have been that given by the doctor of whom an "Admirer of Florence Nightingale" speaks, viz., to put on an apron and do some household work. Because such a woman can afford to have two or three servants as well as a trained nurse attending to all her whims and fancies, are we to be considered unwomanly because we cannot sympathise with her imaginary or sloth-engendered ailment, when we know that there are many with real troubles to whom a conscientious trained nurse would be a perfect Godsend?

"An Admirer of Florence Nightingale" writes: I am not one of those nurses who craves for over-excitement, neither do I own to lack of the true womanly sympathy which I know is so necessary for the noble profession of "nursing." If I were minus this good quality I should never have adopted nursing as a profession. "Sympathy," however, evidently has come to the conclusion that I lack this quality by the tone of her comment, and I, in return, think she lacks "charity." People are very often inclined to judge others as they act themselves. I hope it is not so with "Sympathy." I stick to my old principle still, that private nursing is uninteresting if you do not get acute forms of disease to nurse, such as fever cases and acute forms of lung disease, such as pneumonia, surgical operations, &c. These, I say, are most interesting, and skilled nurses are absolutely necessary in such cases. But I have been on cases for months, and even over a year, where I never had to use much skill; therefore I consider these cases uninteresting to highly-qualified nurses. An untrained nurse could have done the duties I had to perform quite easily. Now, with regard to flirting, I think "Sympathy" and we in the nursing profession have enough outsiders who are only too eager to criticise us, without her chiming in. Nearly every case I go on I am asked the question, "Why do so many young ladies take up nursing as a profession?" "Oh, I think they want to get husbands," is generally said. I cannot answer for nurses as a body. But I for one can assert truly that I did not join the profession with this motive as a stimulant. I must say I had a higher aim in view than husband-hunting. If girls are inclined for familiarity with the opposite sex I consider nursing is not the profession to choose. Evidently "Sympathy" concludes that it is. Now I would give "Sympathy" a word of motherly advice, although I am her junior in the profession, and ask her never to judge too harshly of others, especially if she does not thoroughly understand what they mean.

### FEMALE NURSES AND THE WAR.

"Isle of Wight" writes: "They also serve who only stand and wait." To many of us these words must bring a balm of healing, for are not our hearts throbbing with impatience and longing that we may see "active service," and when we read of the noble work of our sisters by profession at the front, and of Mr. Treves' high praise, we too would "go and do likewise," but we do not appear to be required. Here a thought occurs to me. It may be quite unworkable, but why under the present national stress should not some of us nurses be sent out to take the place of the "orderlies," so as to allow them to go to the front? If, as Mr. Michelli has already suggested, it is a question of expense, could not some of the money subscribed to the war funds with advantage be expended in thus providing female nurses for the military hospitals? Women have proved that they can be brave, as they were at Chieveley, and they are far quicker in thought, and able to make things far more comfortable, than men. Before my mind's eye rise numerous cases where we have had to reduce rooms in absolute chaos to order, and to work with a total absence of anything convenient to use, even having to wash the patient and make jacket poultices with nothing except an old kettle. My experience is that the deft woman's hand is very much missed where men are left to the tender mercies of men. So cannot some of us who are panting to go be sent to take the orderlies' place?

### THE COMMERCIAL ELEMENT IN NURSING.

"One of the Public" writes: I should like to give some experiences bearing upon this question, leaving it to your readers to judge for themselves whether trained nurses are to be included among those who foster the commercial spirit. My mother was a comparative invalid, and was under care and treatment of leading London physicians. Hospital nurses were often deemed necessary, and for many years she was never without one. There was much sympathy and friendship resulting from the services of the nurses, and a great interest taken in nurses and all pertaining to nursing. A few particulars of the circumstances of some of these nurses show the reasons why they worked and why they deserved the proper remuneration for the work. Nurse A. was the daughter of a solicitor having a fair practice, who had died at a comparatively early age, leaving his widow with four sons and three daughters. A severe struggle was before her, left with her young family. With the help of relatives and friends she was enabled to give each of her children a training to enable them to support themselves. The eldest daughter became a high school teacher, the second a nurse. Nurse B. was the daughter of a doctor, a hard-working general practitioner in a country district, where there was hard and incessant work to be done to accrue even a moderate income. She said she could not stay at home when she could be supporting herself, and entered a hospital for training as a nurse, so as to make it possible that her younger brothers and sisters might enjoy educational advantages. Nurse C. was the daughter of a qualified dental surgeon. Both parents died in her infancy; her grandparents provided for her till she was of age to support herself, when she entered a hospital for training as a nurse, so as to be independent and of help to her aged grandparents. Now each one of these girls was working for her living from necessity. The choice of profession was their own, but it was an absolute necessity that they should provide for themselves. They had all come from refined and cultured homes. Is this the commercial spirit complained of? The desire and brave effort of these young gentlewomen to support themselves that they might not be a burden upon their homes and families? To my mind, it is not amongst the nurses themselves that there is evinced any undue tendency to the commercial spirit, but among those who employ nurses, such as nursing homes, nursing establishments, &c. In many of these so-called "homes" the nurses earn over £100 for the institution annually, and personally receive £30 per annum, and have to find their own uniform, and when in the home provide their own washing. In return, they are regarded solely as means of providing money. Let those who complain of a commercial element in nursing remember these facts regarding the life and training of every hospital nurse, when I venture to think they will be more inclined to wonder at the modesty of the scale of remuneration that a trained nurse is asked to receive rather than to carp at the commercial spirit when she asks or adequate salary.

"A Chiel Among Ye" writes: I regret to see the old subject of class distinction in our profession revived. Surely all nurses will acknowledge that, other things being equal, gently-born, refined, and educated women should make the best nurses. But a very short experience of hospital life teaches that natural refinement, intelligence, and devotion to duty are not peculiar to any one class, and that education, in the true sense of the word, is sometimes conspicuous by its absence where we most expect to find it. We do not now, so often as formerly, meet in our ranks the lady, sweet, gentle, and refined, anxious to diffuse "sweetness and light," but without any adequate preparation and utterly incompetent. "*Noblesse oblige!*" Gentlewomen now realise that their preparation and training should be not less, but rather more arduous and complete than that of their poorer sisters. But there are many of the class so well described by "A Matron of Experience"—"Rich women who, from *ennui* or a want of intelligent interest in the pursuits of cultivated people and the ordinary ways of doing good, leaves her surroundings for fresh excitement in a hospital." These are the people alluded to by Ruskin when he says, "Vulgarity shows itself primarily in dulness of heart, in inability to conceive or feel noble character or emotion, in dulness of sense or stupidity." (The whole chapter on this subject might well be printed separately and circulated amongst nurses.) And while the discipline of training and contact with the stern realities of life may be good for such women, I doubt if they elevate the profession, and venture to think that the money value of their services might be given in a more useful form. With regard to the other class mentioned, no doubt some enter the ranks from social ambition, or a desire "to better themselves," but not all or the majority. Indeed, amongst that class the "commercial" spirit, or the instinct of self-preservation, is often most sadly lacking. How often do they remain long in posts where they are overworked and ill-paid because they are absorbed in their work, and find their happiness in the "daily round," the common tasks which are the means of restoring health and hope to countless weary workers; or as private nurses where in many cases they remain on duty long hours, sometimes practically night and day, doing cheerfully whatever appears necessary for the recovery of their patient, who, in some cases, is quite able but unwilling to pay for a second nurse? In the nursing profession there is room and need for the best women of every class, and it will be a sad day when it becomes a preserve for any one class. We have not yet too many women who do

"The work which makes for service, not for fame,
Which buries self, and setteth forth Thy name;
Deeds near, not dreams afar;"

or who can say—if I may alter one word of Ruskin's—"Be it so, with no better reward, no brighter hope, we will be helpers while we may; women just and strong and fearless, and, up to our power, perfect."

### Presentations.

St. Helens Hospital.—Miss Agnes Mellor, who for the past three and a half years has held the post of sister at the St. Helens Hospital, was the recipient of the following handsome presents on the occasion of her resignation to be married to Dr. John Evans, of Carnarvon: Dr. and Mrs. Bates, a drawing-room chair; Dr. and Mrs. Gray, case of dessert knives and forks; Dr. and Mrs. Jackson, Crown Derby afternoon tea service; Dr. and Mrs. Masson, case of silver muffineers and mustard pot; Dr. and Mrs. Reid, silver candlesticks; the matron, a drawing-room chair; the nursing staff (past and present), a Worcester china biscuit drum, pair of silver flower vases, case of silver salt cellars, sugar basin and cream jug and case of afternoon teaspoons. Miss Mellor, by her devotion to duty and her unfailing kindliness, had won the affection and esteem of all who knew her. She carries with her the heartiest congratulations and earnest wishes for her happiness of a wide circle of friends.

Margate Cottage Hospital.—The annual meeting of subscribers to the Margate Cottage Hospital was preceded by an interesting ceremony at the Town Hall on Thursday last week, namely, the presentation of an illuminated resolution and purse of gold to Miss Mary Buxton, who has recently resigned the post of nurse-matron after twelve years' valued and devoted service. The testimonial, which was beautifully illuminated, and framed in black and gold, was as follows: "At a meeting of the president, medical staff, and committee of management, held at the Margate Cottage Hospital on Tuesday, November 22nd, 1899, it was unanimously resolved that the sincerest thanks of the committee of management be accorded to Sister Buxton for her faithful and efficient services as matron for the past twelve years. The committee would further record their high appreciation of her work, and trust many blessings and much prosperity may attend her in her future life." The presentation was made by Mr. W. G. Mercer, J.P., vice-president, and was gracefully acknowledged by Sister Buxton.

Mile End Infirmary.—An interesting ceremony took place in the nurses' recreation room at the Mile End Infirmary last week, when the staff assembled to witness a presentation to Dr. Arthur H. Robinson, the medical superintendent, who is leaving after 15 years' service to take up a similar appointment at the Islington New Infirmary. The presents consisted of a massive and very handsome timepiece in black marble and bronze, bearing a suitable inscription, together with a case of solid silver fruit spoons, gilt and beautifully chased. Dr. Brooks, the assistant medical officer, in making the presentation on behalf of the staff, recalled the long and valuable services Dr. Robinson had rendered to the Mile End Infirmary, making special mention of the founding of the Training School for Nurses, and said they all wished him prosperity and happiness in the new sphere of labour upon which he was about to enter. Dr. Robinson, who was visibly and deeply affected, briefly replied, and thanked the staff for their beautiful gifts, which, he said, would be a lasting remembrance of the good wishes they had expressed towards him.

### For Reading to the Sick.

In the beginning was the Word, and the Word was with God, and the Word was God.—*St. John* i. 1.

Jesus is God;
The solid earth, the ocean broad and bright,
The countless stars, like golden dust,
That strew the skies at night;
The wheeling storm, the dreadful fire,
The pleasant, wholesome air,
The summer's sun, the winter's frost,
His own creations were.

Jesus is God;
Let sorrow come,
And pain and every ill,
All are worth while, for all are means
His glory to fulfil;
Worth while a thousand years of woe
To speak one little word,
If by that "I believe" we own
The Godhead of our Lord.
—*Hymns Ancient and Modern.*

**Reading.**

With all the evil and misery in the world men would not have been able to believe in the Fatherhood of God, meaning His love, without the revelation of it given by Jesus Christ. —*Gore.*

The teaching of Christ, as set forth in the Sermon on the Mount and the parables of the kingdom, has been marvellously realised in human history. At this moment, in all parts of the globe where Christianity has come, there is a vast company of souls living lives of unselfishness, purity, and humility, who, but for Jesus Christ, would be selfish, sensual, proud. At this moment there are numbers of men and women living who have given up all for Christ, and are ready to lay down their lives for His sake. Jesus Christ has produced a new type of humanity, and it is, moreover, a permanent type.

The era of humanity is the era of the Incarnation. The influence of Jesus Christ has radiated from His own Person into the outer world.

It is to Christianity that slavery owes its suppression. It is through Christianity that the rights of the poor are more and more recognised and respected. It is to Christianity that the sick and suffering owe the blessings of loving care in our hospitals. It is by Christianity that women have been raised from degradation to honour. It is through Christianity that the nations are taught to regard each other as brethren.

What is the secret of this wonderful, lasting, and living influence of Jesus Christ? Is it His miracles of love and mercy, His character, His teaching, His death, His resurrection? Is it all these put together? Assuredly not. Combined together, they are inadequate to account for the secret of His mighty influence. But one fact alone can explain the marvel—the fact that He is divine. If Jesus Christ is not God, then all the striking phenomena of His influence are based on misrepresentation and fraud. . . . There is but one solution which adequately explains the problem. The influence of Jesus Christ is the divine influence—Jesus Christ is God.—*Vernon Staley.*

### Where to Go.

New Gallery, Regent Street.—An interesting collection of the works of Rubens, with some of the best pictures of the English School, are to be seen at this gallery.

### Notes and Queries.

The Editor is always willing to answer in this column, without any fee, all reasonable questions, as soon as possible.

But the following rules must be carefully observed:—

1. Every communication must be accompanied by the name and address of the writer.

2. The question must always bear upon nursing, directly or indirectly.

If an answer is required by letter a fee of half-a-crown must be enclosed with the note containing the inquiry.

*Young Probationer.*

(208) Will you kindly tell me if there is any training home, hospital, or infirmary where they take young ladies of 16 years of age as probationers? —*C. C. Y.*

Sixteen is much too young to begin nursing. A very few children's hospitals accept probationers at eighteen.

*Acne.*

(209) Will you kindly inform me what steps I should take to have my name entered in the "Dictionary of Nurses' Names and Careers," which I understand is shortly to be published? 2. Also, I should be grateful if you or any of your correspondents could tell me of a simple remedy or alleviation for acne spots.—*M. E. K.*

1. Send for form to the Editor, "Burdett's Official Nursing Directory," 28 & 29, Southampton Street, Strand. 2. Consult a skin specialist.

*Rheumatism.*

(210) Would you kindly tell me if there is any home at Buxton for ladies of limited means? My sister (21) suffers from rheumatic gout, and the doctor has ordered her to take the baths, but being only a private nurse I cannot afford more than 10s. to 15s. a week for her out of my hard-earned savings.—*Nurse W.*

The Devonshire Hospital and Buxton Bath Charity, Buxton, might suit you. Admission is by a subscriber's letter. Write to the secretary for particulars as to how to obtain one. Or the House of Rest, Hartington House, Buxton. Apply hon. secretary. This is open to ladies from April 1st Terms from 12s. 6d. a week.

*Aseptics.*

(211) Will you kindly tell me the difference between "aseptic" and "antiseptic," *i.e.*, what difference is there in boiling and the carbolic spray? 2. Also, how much should each pint of water contain of the antiseptic when ordered 1 in 1,000. Being accustomed to using carbolic 1 in 20 or 40 I am puzzled over the above.—*E. M. I.*

Aseptic means without putrefaction, and the term is applied to surfaces, &c., so perfectly clean that they are absolutely free from the presence of germ. Antiseptic, meaning against putrefaction, is a term applied to dressings, lotions, &c., possessing the property of destroying germs. 2. Moist heat above boiling point and carbolic in any form are both antiseptics, but one can be used where the other would be injurious.

*Open Air Treatment.*

(212) Will you kindly let me know what free hospitals there are for the open air treatment of consumption?—*M. H.*

The charges at the newly-opened sanatoria for the open air treatment of consumption are heavy. The Consumption Hospital, Brompton; the North London Hospital for Consumption, Mount Vernon, Hampstead, &c., are free.

*South Africa.*

(213) Is it likely I could get work in any of the hospitals or hospital ships in South Africa? I am not certificated, but have had a good deal of experience, and I have good testimonials. To whom should I apply?— *H. L. M.*

It is hopeless for any but trained nurses to obtain work in South Africa.

*Ruptured Perinæum.*

(214) Is it usual for a certificated midwife to stitch a ruptured perinæum? A nurse was surprised to find that the midwife attending such a case did not call in a doctor when this occurred.—*A. L.*

The suture of a lacerated perinæum is a surgical proceeding, which ought to be done by a surgeon. But it is an emergency which may occur where surgical help is not available, and as it is a thing which may happen to anyone, even in otherwise simple cases, and as immediate suture is in most cases important, we think that a midwife should be prepared to do her best in the absence of a surgeon. Nevertheless, a surgeon should be sent for, for all is not over when the sutures are put in. The necessity for action in an emergency is no excuse for the midwife continuing the treatment after the emergency is over.

*Resignation.*

(215) Suppose a nurse give a month's notice on, say, January 10th, would she be expected to go on duty either for the whole or part of February 10th?—*Nurse.*

She would be expected to perform her duties on February 10th. In giving such notice, however, it is customary to arrange the hour of departure with the matron so that each may consult the convenience of the other.

*Standard Books of Reference.*

"The Nursing Profession: How and Where to Train." 2s. net.
"The Nurses' Dictionary of Medical Terms." 2s. 6d. net.
"Burdett's Series of Nursing Text-Books." 1s. each.
"A Handbook for Nurses." (Illustrated.) 5s.
"Nursing: Its Theory and Practice." New Edition. 3s. 6d.
"Helps in Sickness and to Health." Fifteenth Thousand. 5s.

All these are published by The Scientific Press, Ltd., and may be obtained through any bookseller or direct from the publishers, 28 & 29, Southampton Street, London, W.C.

